# Steroid Derivatives as Potential Antimicrobial Agents against *Staphylococcus aureus* Planktonic Cells

**DOI:** 10.3390/microorganisms8040468

**Published:** 2020-03-25

**Authors:** Adriana Vollaro, Anna Esposito, Eleni Antonaki, Vita Dora Iula, Daniele D’Alonzo, Annalisa Guaragna, Eliana De Gregorio

**Affiliations:** 1 Department of Molecular Medicine and Medical Biotechnology, University of Naples Federico II, Via S. Pansini 5, 80131 Naples, Italy; vollaroadriana@libero.it (A.V.); antele2006@hotmail.it (E.A.); 2 Department of Chemical Sciences, University of Naples Federico II, Via Cintia, 80126 Naples, Italy; anna.esposito5@unina.it (A.E.); daniele.dalonzo@unina.it (D.D.); 3 Complex Operative Unit of Clinical Pathology, “Ospedale del Mare-ASL NA1 Centro”, 80147 Naples, Italy; dora.iula@gmail.com

**Keywords:** antimicrobial activity, *Staphylococcus aureus*, steroids, deflazacort (DFZ), multidrug pathogens, anti-virulence agent, quantitative real-time PCR, checkerboard assay

## Abstract

In this work, the antibacterial activity of deflazacort and several of its synthetic precursors was tested against a panel of bacterial pathogens responsible for most drug-resistant infections including *Staphylococcus aureus, Enterococcus* spp., *Acinetobacter baumannii*, *Pseudomonas aeruginosa*, *Klebsiella pneumoniae*, *Escherichia coli*, and *Enterobacter* spp. The derivative of deflazacort, PYED-1 (pregnadiene-11-hydroxy-16α,17α-epoxy-3,20-dione-1) showed the best antibacterial activity in a dose-dependent way. We focused on the action of PYED-1 against *S. aureus* cells. PYED-1 exhibited an additive antimicrobial effect with gentamicin and oxacillin against the methicillin-resistant *S. aureus* isolate 00717. In addition to its antimicrobial effect, PYED-1 was found to repress the expression of several virulence factors of *S. aureus*, including toxins encoded by the *hla* (alpha-haemolysin), *hlb* (beta-haemolysin), *lukE-D* (leucotoxins E-D), and *sea* (staphylococcal enterotoxin A) genes, and cell surface factors (*fnbB* (fibronectin-binding protein B) and *capC* (capsule biosynthesis protein C)). The expression levels of autolysin *isaA* (immunodominant staphylococcal antigen) were also increased.

## 1. Introduction

Hospital-acquired infections represent a major problem in high or upper-middle income countries, with an incidence rate of 5% in the United States and 7.1% in Europe [1]. The therapeutic options for infections caused by multidrug-resistant (MDR) pathogens are often extremely limited. Despite intensive searches for new antimicrobial agents, there are, to date, a few active candidates [2], and new antibacterial substances acting through non-conventional mechanisms are needed [3]. 

**Figure 1:**
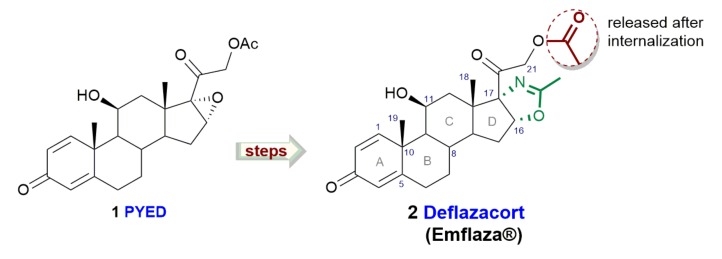
Deflazacort and its synthetic precursor PYED-1 (pregnadiene-11-hydroxy-16α,17α-epoxy-3,20-dione-1).

Steroids, a family of naturally-occurring secondary metabolites characterized by a four-fused ring structure, have exhibited a wide range of biological activities and have been shown to function as antitumoral, antiviral, antibacterial, and antioxidant agents [4]. The heterocyclic corticosteroid deflazacort (DFZ, Figure 1), an oxazoline-derivative of prednisolone, has been recently approved for the treatment of Duchenne dystrophy [5] showing high efficacy and good tolerability.

In the frame of a study devoted to the development of a novel and convenient synthetic strategy for the preparation of DFZ, we recently found that a DFZ synthetic precursor called PYED-1 (pregnadiene-11-hydroxy-16α,17α-epoxy-3,20-dione-1)**,** exhibited a good antibacterial activity against *Staphylococcus aureus* ATCC 29213 and *Acinetobacter baumannii* ATCC 17978 without showing cytotoxicity [6]. In addition, we demonstrated that PYED-1 has a weak effect against *Stenotrophomonas maltophilia* clinical isolates but at sub-inhibitory concentrations it inhibits biofilm formation of the reference *S. maltophilia* K279a strain [7].

Here, an improved protocol for the synthesis of PYED-1 has been reported, along with the evaluation of the in vitro antimicrobial activity of PYED-1 and some steroidal synthetic precursors and analogues of DFZ against a panel of bacterial pathogens responsible for most drug-resistant infections, including *Staphylococcus aureus, Enterococcus* spp., *Acinetobacter baumannii*, *Pseudomonas aeruginosa*, *Klebsiella pneumoniae*, *Escherichia coli*, and *Enterobacter* spp. We focused our attention on the activity of PYED-1 against *S. aureus.* This microorganism causes a variety of infections that range from topical to life-threatening infections and represents a major problem in both the hospital and community settings [8,9]. *S. aureus* has rapidly-acquired resistance to many antibiotic drug classes, causing infections that are difficult to eradicate [10]. Additive antimicrobial activity with conventional antibiotics against *S. aureus* cells by checkerboard microdilution method was assessed. Finally, real-time PCR was performed to evaluate the effect of PYED-1 on *S. aureus* genes involved in the virulence factor production. 

## 2. Materials and Methods 

### 2.1. Chemicals and Reagents

All chemicals and solvents were purchased with the highest degree of purity (Sigma-Aldrich, Alfa Aesar, VWR) and used without further purification. The reactions were monitored by TLC (precoated silica gel plate F254, Merck) and the products were detected by exposure to ultraviolet radiation, iodine vapor, and chromic mixture. The purity of the compounds was determined by CHNS analysis and was ≥95% in all cases. NMR spectra were recorded on NMR spectrometers operating at 400 MHz (Bruker DRX, Bruker AVANCE) using CDCl_3_ solutions. Coupling constant values (*J*) were reported in Hz. Chemical synthesis and structural characterization of compounds **3** to **7** has been realized as previously reported [6].

2-((4aS,4bR,5S,6aS,6bS,7aR)-4b-bromo-5-hydroxy-4a,6a-dimethyl-2-oxo-2,4a,4b,5,6,6a,7a,8,8a,8b,9,10-dodecahydro-6bH-naphtho [2’,1’:4,5]indeno[1-b]oxiren-6b-yl)-2-oxoethyl acetate (compound 10). Phthalic anhydride (0.13 g, 0.86 mmol) was added in one portion to a stirred solution of compound 9 (provided by Symbiotec Pharmalab PVT) in dichloromethane (DCM) 2.0 mL at room temperature. After few minutes, phthalic anhydride was completely dissolved to give a yellow clear solution. At the same temperature 50% aqueous H_2_O_2_ was then added dropwise in 2h and the mixture was then heated to reflux temperature, until formation of a white precipitate was observed. The reaction mixture was stirred at the in-refluxing DCM for 24 h. Water and solid sodium bicarbonate (NaHCO_3_) were added to the cooled reaction mixture until pH 7 was reached, and then the mixture extracted with dichloromethane (CH_2_Cl_2_). The organic layer was washed with brine while the aqueous phase was again extracted with chloroform/methanol (CHCl_3_/MeOH, 9/1). The resulting organic layers were washed with brine. All the organic phases were combined, dried with sodium sulfate anhydrous (Na_2_SO_4_) and concentrated under reduced pressure to give a yellow solid. Ethyl acetate was added to the crude residue. The resulting precipitate was decanted, and the orange mother liquors removed. This operation was repeated until a white powder was obtained. All the mother liquors previously obtained were combined and concentrated. The resulting precipitate was decanted, separated from the mother liquors and repeatedly washed with ethyl acetate until to obtain epoxide derivative 10 as a white powder (85 mg, 82%yield). ^1^H NMR (400 MHz): δ 1.41 (s, 3H, H-18), 1.48-1.55 (m, 1H, H-14), 1.70 (s, 3H, H-19), 1.71-1.82 (m, 2H, H-7), 1.94-2.05 (m, 3H, H-12a, H-15), 2.15 (s, 3H, H-23), 2.16–2.25 (m, 1H, H-8), 2.36–2.49 (m, 2H, H-12b, H-6a), 2.55–2.68 (m, 1H, H-6b), 3.85 (s, 1H, H-16), 4.59 (d, J = 13.4, 1H, H-21a), 4.67 (d, J = 13.4, 1H, H-21b), 4.76 (bs, 1H, H-11), 6.07 (bs, 1H, H-4), 6.32 (d, J = 10.1, 1H, H-2), 7.21 (d, J = 10.1, 1H, H-1). ^13^C NMR (100 MHz): 18.3, 20.4, 24.9, 27.0, 28.3, 30.4, 33.5, 37.3, 39.3, 42.1, 50.2, 61.1, 65.7, 70.5, 75.9, 85.3, 125.1, 129.3, 152.3, 165.5, 170.4, 186.2, and 198.9. Anal. calcd for C_23_H_27_BrO_6_: C, 57.63; H, 5.68; Br, and 16.67. Found: C, 57.75; H, 5.66; and Br, 16.62.

2-((4aR,5S,6aS,6bS,7aR)-5-hydroxy-4a,6a-dimethyl-2-oxo-2,4a,4b,5,6,6a,7a,8,8a,8b,9,10-dodecahydro-6bH-naphtho[2’,1’:4,5]indeno[1,2-b]oxiren-6b-yl)-2-oxoethyl acetate (PYED-1) was prepared from compound 10 by treatment with tributyltin hydride (Bu_3_SnH) and azobisisobutyronitrile (AIBN) according to the procedure previously reported [6].

### 2.2. Biological Screening

#### 2.2.1. Bacterial Strains and Growth Conditions

Bacterial clinical isolates belong to a collection previously established at the Department of Molecular Medicine and Medical Biotechnology, University of Naples Federico II. In accordance with European regulations, each isolate was associated with a unique ID and subsequently anonymized. Epidemiological features of strains were in accordance to previous publications [11,12,13,14]. No ethical approval was required for the study because there was no access to patient data. Antimicrobial susceptibility to conventional antibiotics of all isolates was determined using the VITEK 2 system (BioMérieux) and/or the Phoenix microbiology system (BD Diagnostics) and the results were interpreted in accordance with the European Committee on Antimicrobial Susceptibility Testing [15] guidelines. The minimal inhibitory concentration (MIC) of colistin of *A. baumannii* isolates was determined by broth microdilution assay according to Clinical Laboratory Standards Institute guidelines [16]. The MIC of oxacillin was determined by broth microdilution method in cation-adjusted Mueller–Hinton broth (CA-MHB) supplemented with 2% NaCl. The plates were incubated at 35 °C and the MIC was determined after 24 and 48 h of incubation. The presence of the staphylococcal cassette chromosome *mec* (SCC*mec*) in methicillin-resistant *S. aureus* (MRSA) isolates was assessed using the Xpert MRSA kit (Cepheid, Sunnyvale, CA). The kit contains primers and probes for the detection of the *spa*, *mecA*, and SCC*mec* sequences inserted into the SA chromosomal *attB* site. The Xpert MRSA assay was performed using a GeneXpert Dx system and the analysis was performed as per the manufacturer’s protocol. 

#### 2.2.2. Antimicrobial Activity of Steroidal Compounds

MIC values of steroidal compounds against planktonic bacteria were examined by a broth microdilution method previously described [17]. Briefly, compounds were dissolved in dimethyl sulfoxide (DMSO) to the concentration of 50 mg/mL. Two-fold serial dilutions ranging from 2 μg/mL to 1000 mg/mL of the compounds were prepared in triplicate and placed into a polystyrene 96-well plate. Bacterial cell suspensions were prepared at 0.5 McFarland standard using BD PhoenixSpec™ nephelometer and were subsequently diluted in cation-adjusted Mueller–Hinton broth (CA-MHB) to final culture density of approximately 5 × 10^6^ colony forming unit (CFU)/mL. One hundred microliter of bacteria (5 x 10^5^ CFU) were then added to the microtiter plates containing 100 μL of serial dilutions of steroidal compounds. Only CA-MHB was added in negative control wells. Wells with no compounds were used on each plate as positive growth control. Plates were incubated at 37 °C for 18–24 h under shaking (300 rpm). To evaluate microbial growth, the optical density at 595 nm was measured by using a microplate reader (Bio-Rad Laboratories S.r.l.). The effects of DMSO concentrations, ranging from 0.1% to 1%, on bacteria growth kinetics were separately tested. To calculate the minimum bactericidal concentration (MBC), bacterial suspensions from MIC assay microtiter wells were diluted in PBS and spot-plated on TSA plates to count colonies after incubation at 37 °C for 18 h. The MBC was determined as the lowest concentration of substance, which produced ≥99.9% killing (≥3log10) after 24 h of incubation as compared to the colony count of the starting inoculum. All tests were performed in triplicate and repeated three times. 

#### 2.2.3. Time Kill Assay

The killing kinetics of PYED-1 at 1 x, 2 x, and 4 x MIC were determined against *S. aureus* ATCC 29213 strain, as previously described [18]. Tubes containing CA-MHB with different concentrations of PYED-1 were inoculated with *S. aureus* ATCC 29213 to a density of approximately 5 x 10^6^ CFU/mL and incubated at 37 °C under shaking (300 rpm). The tube without PYED-1 was a growth control. Viable bacterial counts were performed after 0, 1, 2, 3, 4, 5, 7, and 24 h incubation by plating serial 10-fold dilutions of broth cultures onto TSA plates.

#### 2.2.4. Checkerboard Assay

A checkerboard method in 96-well microtiter plates containing CA-MHB was used to test the effects of the interactions of PYED-1 with either oxacillin or gentamicin against methicillin-resistant *S. aureus* (MRSA). In brief, PYED-1 and each tested antibiotic were serially diluted in microtiter plate wells along the y and x axes. Checkerboard plates with a concentration of 10^6^ CFU/mL of the tested bacteria were incubated overnight at 37 °C. To evaluate microbial growth, the optical density at 595 nm was measured by using a microplate reader (Bio-Rad Laboratories S.r.l.). The effect of the interactions of PYED-1 with each of the tested antibiotics was quantified by calculating the fractional inhibitory concentration (FIC) index as follows: FIC index = FIC of PYED-1 + FIC of antibiotic, where FIC of PYED-1 (or antibiotic) is the ratio of MIC of PYED-1 (or antibiotic) in combination and MIC of PYED-1 (or antibiotic) alone. The following intervals of FIC index were used to interpret the experimental outcomes: ≤0.5, synergistic; >0.5 to ≤1.0, additive; >1.0 to ≤2.0, indifferent; and >2.0, antagonistic effects [19]. All experiments were repeated three times. 

#### 2.2.5. RNA

Total RNA was isolated from *S. aureus* ATCC 29213 cells grown overnight in CA-MHB were diluted to an OD_600_ of 0.05 and grown at 37 °C at 200 rpm to an OD_600_ of 0.3–0.4. The culture was subsequently split into two tubes, treated with either PYED-1 at the concentration of 4 μg/mL or 0.008% DMSO, and incubated at 37 °C at 200 rpm for a further 3 h. Two volumes of RNAprotect Bacteria Reagent was added to the cell suspensions and incubated for 5 min at room temperature. Next, the cell suspensions were centrifuged at 5000× *g* for 10 min and the supernatant was decanted. RNA was purified according to the previously-reported method [20] with minor modifications. Bacterial cells were resuspended in 200 μL of 20 mg/mL proteinase K and 200 μL TE buffer (30 mM Tris HCl, 1 mM EDTA, pH 8.0) containing 20 mg/mL lysozyme and 12.5 μg/mL lysostaphin and incubated at 37 °C for 1 h. The resulting protoplasts were used for RNA isolation with the RNeasy Mini Kit, following recommendation of the manufacturer (Qiagen, Germany). Residual DNA were removed with DNase Max Kit (Qiagen). RNA was quantified using a Nano-drop instrument (Thermo Fisher). 

#### 2.2.6. RT-PCR

Total RNA was reverse transcribed into cDNA using QuantiTect Reverse Transcription Kit (Qiagen), according to the manufacturer’s protocol. The RT-PCR was performed as previously described [13], using a SYBR Green master mix (Applied Biosystems). The primer pairs used in PCR experiments are reported in Table 1. 

The *rpoB* gene was used as the housekeeping control to normalize the expressions of genes of interest. RNA samples not treated with reverse transcriptase were routinely included as no template controls. Changes in transcript levels were determined using the 2−^ΔΔCT^ method [21]. RNA expression levels were determined by using three independent cultures and all analyses were performed in triplicate.

#### 2.2.7. Statistical Analysis

Each experiment was performed in triplicate and repeated at least three time on different days. Arithmetic means and standard deviations were used to statistically analyze continuous variables. Student’s t test was used to determine statistical differences between two means.

## 3. Results

### 3.1. Chemistry

**Figure 2:**
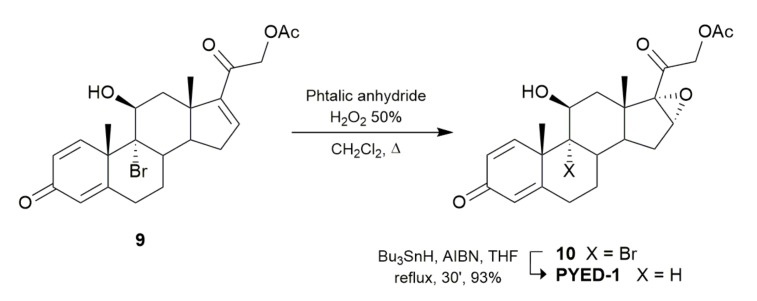
Synthetic transformations from brominated compound 9 to epoxide PYED-1.

To deeply study the biological potential of PYED-1, an improved protocol for its preparation was first designed (Figure 2). 

As previously described [6], C16–C17 double bond oxidation of 9-bromotriene acetate (compound 9) with *meta*-chloroperbenzoic acid (*m*CPBA) led to the corresponding PYED-1, albeit with uncomplete conversion (47% yield). With the aim to improve the reaction yield, the use of a different organic peroxy acid, i.e., monoperphthalic acid, was investigated [22]. As shown in Figure 2, treatment of compound 9 with phthalic anhydride in the presence of 50% of aqueous H_2_O_2_ in refluxing DCM gave, after 24 h, the corresponding brominated epoxide (compound 10) in better yields (82%). Debromination of this latter was then accomplished by treatment with Bu_3_SnH and AIBN in refluxing tetrahydrofuran (THF) affording the pure PYED-1 in 93% yield. 

### 3.2. Antimicrobial Activity of a Panel of Steroid Derivatives

**Figure 3:**
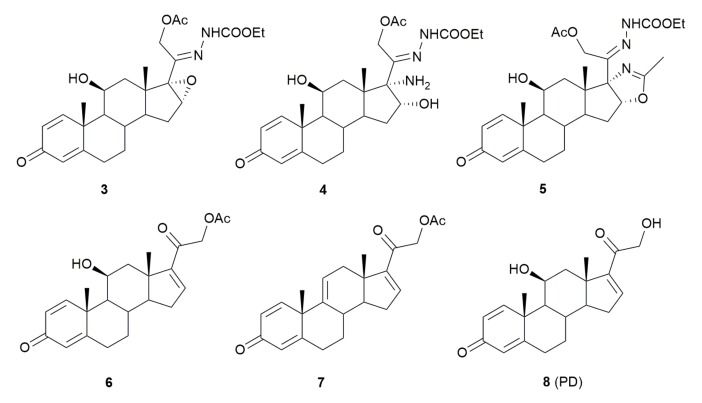
Deflazacort (DFZ) glucocorticoid precursors and analogues.

PYED-1 was shown to be effective against both *S. aureus* and *A. baumannii* [6]. Here, we have chosen to extend the antimicrobial activity study of DFZ and its precursors (Figure 3) against a panel of bacterial pathogens including *S. aureus, Enterococcus* spp., *A. baumannii*, *P. aeruginosa*, *K. pneumoniae*, *E. coli*, and *Enterobacter* spp. 

The MIC of all steroid derivatives was determined by broth microdilution assay. The antibacterial activity of steroid derivatives against selected bacteria varied significantly, MIC values ranging from 4 to 750 μg/mL (Table 2). 

PYED-1 proved to be the most effective growth inhibitor, with 4 and 16 μg/mL MIC values against *Enterococcus faecalis* and *S. aureus* respectively, instead shown a 16 μg/mL MIC values against *A. baumannii* and 128 μg/mL against both *E. coli* and *Pseudomonas aeruginosa*. No antimicrobial activity was found for DFZ and compound 4. The intermediates 3 and 5 showed only a weak antimicrobial activity against *E. faecalis* and *S. aureus*, and only the compound 3 showed a weak antimicrobial activity against *P. aeruginosa*. All compounds did not exert any appreciable inhibition of the other Gram-negative bacteria tested, *K. pneumoniae* and *E. aerogenes*, up to the concentration of 1000 mg/mL. To examine whether the inhibition of bacterial growth could be related to the DMSO used to dissolve the compounds tested, the bacterial growth was measured in the presence of increasing concentrations of DMSO. No growth differences were observed in the presence of any of the DMSO concentrations used (data not shown).

With the aim to identify which functionalities could be responsible for the antimicrobial activity of PYED-1 we evaluated the biological potential of compounds 6, 7, and 8, which share common residues with PYED-1. Compound 6, in which the epoxy function at C16–C17 positions was replaced by a double bond (Figure 3), exhibited antibacterial activity against *S. aureus*, *E. faecalis*, *A. baumannii*, *P. aeruginosa,* and *E. coli* strains, but at much higher concentration than PYED-1 (Table 2). In contrast, both compound 7, in which the hydroxyl function at C10 position is absent, and compound 8 (PD) in which the acetyl group at 21 hydroxyl function is missing, functioned only at concentration of 1 mg/mL, or more (Table 2). Data directly imply that the epoxy function on C16–C17 positions of the steroidal scaffold, the C10 hydroxyl group, and the acetyl residue are all crucial for the antimicrobial activity exhibited by PYED-1.

To test whether PYED-1 was also active against clinical strains, we selected for each tested species 8 to 11 clinical isolates exhibiting different antibiotic resistance profiles. For all species, the results obtained with clinical isolates were comparable to those obtained using PYED-1 against the reference ATCC strain (Table 3). 

### 3.3. Activity of PYED-1 against S. aureus 

**Figure 4:**
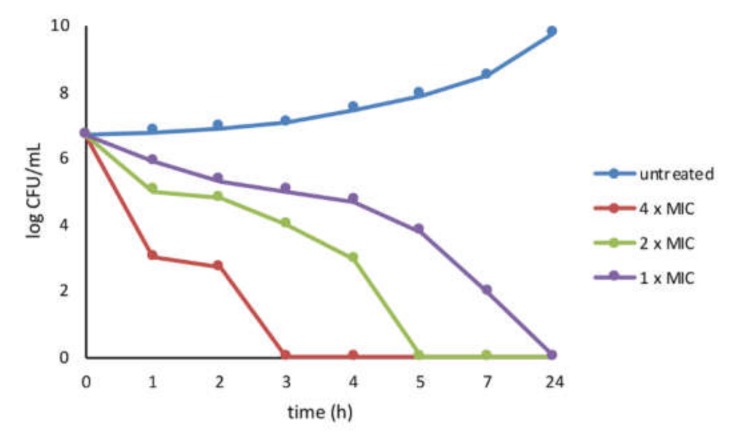
Killing kinetics for *S. aureus* following treatment with the PYED-1. Growth kinetics were monitored following exposure to PYED-1 at 1 x MIC, 2 x MIC, and 4 x MIC.

We focused our attention to the inhibitory effect of PYED-1 against *S. aureus*. The inhibition of bacterial growth by PYED-1 was characterized by means of time–kill assays for the *S. aureus* ATCC 29213. *S. aureus* cells treated with PYED-1 at 4 x MIC (64 μg/mL) and 2 x MIC (32 μg/mL) were killed at 3 and 5 h, respectively. After 7 h exposure to 1 x MIC (16 μg/mL), the amount of *S. aureus* cells dropped to 2 × 10 CFU/mL. After 24 h, there was a total growth inhibition (Figure 4). 

Antibiotic combination therapy is frequently used as a possible method of outmaneuvering recalcitrant bacterial pathogens, to improve the antibiotic efficiency as well as to reduce antibiotic doses used [23]. Synergy experiments using combinations of PYED-1 with conventional antibiotics were conducted in order to potentiate or restore the antibacterial activity of currently available antibiotics against *S. aureus*, a pathogen that is difficult to treat due to the rising number of drug- resistant strains. PYED-1 antibacterial activity was tested in combination with gentamicin and oxacillin against MRSA 00717, an isolate resistant to the selected antibiotics. The MIC value of PYED-1 was 16 μg/mL while the MIC value of gentamicin and oxacillin was 256 μg/mL and 16 μg/mL, respectively. In the checkerboard dilution test, PYED-1 markedly lowered the MICs of gentamicin (from 256 to 16 μg/mL) and oxacillin (from 16 to 1 μg/mL) against the MRSA 00717 isolate. The combination of PYED-1 with either gentamicin or oxacillin had an additive effect, as the FIC values were 0.5625 and 0.5156, respectively (Table 4). 

### 3.4. Transcriptional Changes Induced by PYED-1 in S. aureus

To investigate the anti-virulence activity of PYED-1 the expression levels of known *S. aureus* virulence and regulatory genes [24] were investigated by qRT-PCR (Table 5).

RNA was extracted from exponential *S. aureus* cells (5 × 10^8^ CFU/mL) untreated and treated at sub-MIC concentration (4 μg/mL) of PYED-1 for 3 h. No growth differences between treated and untreated cells were observed. The expression of *spa* (surface protein A) and *clfB* (clumping factor B) genes was not affected, but significant downregulation of *fnbB* (fibronectin-binding protein B) and *capC* (capsule biosynthesis protein C) genes was observed. As shown in Table 5, the levels of toxin genes *hla* (alpha-haemolysin), *hlb* (beta-haemolysin) *lukE-D* (leucotoxins E-D), and *sea* (staphylococcal enterotoxin A) were notably reduced. Downregulation of the transcript levels of *agrA* (accessory gene regulator protein A), *saeR* (response regulator SaeR), and *sigB* (RNA polymerase sigma factor B) genes after PYED-1 treatment by a factor of 2.29, 8.66, and 2.11, respectively, was also noted. Significant upregulation of *isaA* (immunodominant staphylococcal antigen) gene was observed. The expression of *lytM* (peptidoglycan hydrolase) and *aur* (aureolysin) genes was not affected (Table 5).

## 4. Discussion

PYED-1 is a promising novel antimicrobial agent [6]. Here we extended our initial studies on the antimicrobial activity of this compound, by testing its efficacy against a panel of Gram-negative and Gram-positive pathogens, including *S. aureus, Enterococcus* spp., *A. baumannii*, *P. aeruginosa*, *K. pneumoniae*, *E. coli*, and *Enterobacter* spp. PYED-1 exerted a good inhibitory activity against *E. faecalis*, *S. aureus,* and *A. baumannii*, low to moderate activity against *E. coli* and *P. aeruginosa*, while it did not exert any appreciable inhibition against *K. pneumoniae* and *E. aerogenes* ( Table 2 and Table 3). A weak effect was also observed in a previous report against *S. maltophilia* [7].

According to the European Centre for Disease Prevention and Control, *S. aureus* is one of the most difficult-to-treat pathogens due to the increasing number of drug-resistant strains [10]. The time-kill curve analysis against *S. aureus* demonstrated a concentration-dependent rapid bactericidal activity of PYED-1 (Figure 4). In line with this, we have previously shown that PYED-1 exhibited bactericidal activity against *S. maltophilia*, and that the permeabilization of the bacterial membrane might contribute to the effect [7]. Our study demonstrated additivity of PYED-1/gentamicin and PYED-1/oxacillin combinations by checkerboard assay. Data also revealed that PYED-1 has the potential to restore the effectiveness of oxacillin against MRSA strains. This result is an important finding because restoring activity to conventional antibiotics using combinations would enable the clinical use of conventional antibiotics for the treatment of *S. aureus* infections.

Many studies suggested that drugs targeting virulence factors (e.g., enterotoxins, hemolysins, and adhesins) represent an alternative approach to treat infections caused by MDR bacteria [25]. Intriguingly, we observed that PYED-1 impinges on the expression levels of several virulence factors and regulatory genes involved in *S. aureus* virulence (Table 5). The pathogenicity of *S. aureus* depends on the production of numerous extracellular virulence factors. *S. aureus* produces a series of hemolysins including α- and β-hemolysins. Alpha-hemolysin is the most studied pore-forming toxin and it plays a significant role in skin and soft tissue infections in animal models of staphylococcal infection [26]. Highly virulent *S. aureus* strains can produce up to five different bicomponent leukotoxins, by which *S. aureus* targets and kills neutrophils. The pore-forming leukocidin LukE-D is a critical virulence factor involved in *S. aureus* bloodstream infection [27]. 

The regulation of virulence factors induced by an antibiotic may result in either worsening or mitigation of the infection. For diseases caused by *S. aureus,* the regulation of secretion of toxins, and the selection of antibiotic according to its ability to affect these properties could be very important for the outcome of *S. aureus* diseases. At suboptimal concentrations, linezolid and clindamycin significantly inhibits the production of α-hemolysin and staphylococcal enterotoxins (A and B). Investigation of the expression of several toxins of *S. aureus* in the presence of PYED-1 showed protein A [28,29]. In contrast, β-lactams and glycopeptides induce the expression of α-hemolysin, enterotoxins and toxic shock syndrome toxin-1 [30]. We found that the levels of *hla*, *hlb lukD*, *lukE*, and *sea* genes were notably reduced (Table 5). The combination of PYED-1, which downregulates toxins expression, with β-lactams antibiotics, is able to induce expression of α-hemolysin and enterotoxins at subinhibitory concentrations and may therefore be highly beneficial for the treatment of *S. aureus* infections. 

In *S. aureus*, the expression of several virulence genes is regulated by two-component systems, such Agr operon and SaeRS, the DNA-binding protein SarA and the alternative sigma factor SigB. These regulators coordinately control *hla* expression [31,32]. SaeRS also activates the expression of the leukotoxins LukE-D [33]. We observed that treatment with PYED-1 resulted in reduced expression of *agrA*, *saeR,* and *sigB* genes (Table 5). This nicely explains the downregulation of the transcript levels of controlled *hla*, *hlb*, *lukD*, *lukE*, and *sea* genes. These results are in accord with literature data. Duan et al. [34] showed subinhibitory concentration of resveratrol decreased the expression of *hla* and inhibited the regulation of *saeRS*. Subinhibitory concentrations of thymol significantly inhibited the transcription of *hla*, *sea,* and *seb* in *S. aureus* and acted partly through the agr locus [35].

Many antibiotics can affect the expression of *S. aureus* surface proteins. Tigecycline treatment downregulated the expression of the *cap* genes, which mediate the synthesis of the capsule polysaccharide [36]. Oxacillin, moxifloxacin, and linezolid led to the development of a hyper-adhesive phenotype due to an increase in *fnbA/B* transcription [37]. The presence of PYED-1 also reduced the level of other virulence-associated genes in *S. aureus*, such as *fnbB* and *capC* genes in planktonic cells (Table 5), influencing the adherence properties of *S. aureus*. Since microbial adherence is the initial step of *S. aureus* pathogenicity, the ability of PYED-1 to affect this property may be an important factor for attenuation of bacterial infections. 

Autolysins have been implicated in many cellular functions [38]. IsaA is a lytic transglycosylase involved in cellular elongation, septation, recycling of muropeptides, and pore formation [39,40]. The expression levels of the autolysin gene *isa*A were notably upregulated after PYED-1 treatment (Table 5). Overexpression of *isa*A could lead to a reduction in biofilm formation, a major virulence factor involved in *S. aureus* pathogenicity. These results are in accord to Payne et al. [41] that showed tannic acid inhibits the formation of biofilms by increasing the extracellular level of IsaA.

In light of the results obtained with *S. aureus*, further research on the anti-virulence activity of PYED-1 against other multidrug pathogens is merited. 

## 5. Conclusions

The results of the present study revealed that PYED-1, alone or in combination with antibiotics, exerts a strong antimicrobial activity against *S. aureus* growth. PYED-1 has also the potential to restore the effectiveness of oxacillin against MRSA strains. Moreover, the finding that PYED-1 affects the expression levels of *S. aureus* virulence genes make this drug a promising candidate for the treatment of *S. aureus* infectious diseases. 

## Figures and Tables

**Table 1:** Gene target list and oligonucleotide sequences.

Gene	Primer Sequence
*agr*A Fw	TGCGAAGACGATCCAAAAC
*agr*A Rv	TTTAGCTTGCTCAAGCACCTC
*aur* Fw	GATGGTCGCACATTCACAAG
*aur* Rv	CGCCTGACTGGTCCTTATATTC
*cap*C Fw	CATCCAGAGCGGAATAAAGC
*cap*C Rv	CGGAAATACCCGCTAATGAC
*clf*B Fw	TTATGGTGGTGGAAGTGCTG
*clf*B Rv	TGGACTTGGTTCTGGATCTG
*fnb*B Fw	GAACATGGTCAAGCACAAGG
*fnb*B Rv	ACGCCATAATTACCGTGACC
*hla* Fw	TCTTGGAACCCGGTATATGG
*hla* Rv	AGCGAAGTCTGGTGAAAACC
*hlb* Fw	GTGCCAAAGCCGAATCTAAG
*hlb* Rv	ATCAGCGCGTTTATATTGTCC
*isa*A Fw	TCCGACAAACACTGTTGACC
*isa*A Rv	AATCCCCAAGCACCTAAACC
*lyt*M Fw	ACGGTGTCGACTATGCAATG
*lyt*M Rv	ATTGCCGCCACCATAGTTAC
*luk*D Fw	GTACTTAAGGCAGCCGGAAAC
*luk*D Rv	CGCCCCAATAAAACTGTGAG
*luk*E Fw	ATGGGGTGTTAAAGCAAACG
*luk*E Rv	TCTCTTGCTGAACCTGTTGG
*rpo*B Fw	ACAACCACTTGGCGGTAAAG
*rpo*B Rv	ATGCTTCAAGTGCCCATACC
*sae*R Fw	CCAAGGGAACTCGTTTTACG
*sae*R Rv	ACGCATAGGGACTTCGTGAC
*sea* Fw	ATTGCCCTAACGTGGACAAC
*sea* Rv	TGCTCCCTGCAATTCAGAC
*sig*B Fw	TGATCGCGAACGAGAAATC
*sig*B Rv	ATTGCCGTTCTCTGAAGTCG
*spa* Fw	AGATGACCCAAGCCAAAGTG
*spa* Rv	CTTTCGGTGCTTGAGATTCATT

**Table 2:** Minimum inhibitory concentration (MIC) (μg/mL) and minimum bactericidal concentration (MBC) (μg/mL) values of PD, DFZ and its precursors against a panel of Gram-negative and Gram-positive pathogens.

Bacteria	Compound															
	PYED-1		2 (DFZ)		3		4		5		6		7		8 (PD)	
	MIC	MBC	MIC	MBC	MIC	MBC	MIC	MBC	MIC	MBC	MIC	MBC	MIC	MBC	MIC	MBC
*Staphylococcus aureus* ATCC 29213	16	16	>1000	>1000	375	>1000	>1000	>1000	750	>1000	256	512	1000	1000	>1000	>1000
*Enterococcus faecalis* ATCC 29212	4	16	>1000	>1000	187	>1000	>1000	>1000	375	>1000	128	256	1000	1000	>1000	>1000
*Acinetobacter baumannii* ATCC 17978	16	16	>1000	>1000	>1000	>1000	>1000	>1000	>1000	>1000	512	512	1000	1000	>1000	>1000
*Pseudomonas aeruginosa* ATCC 27859	128	1000	>1000	>1000	750	>1000	>1000	>1000	>1000	>1000	1000	1000	1000	1000	>1000	>1000
*Escherichia coli* ATCC 25922	128	128	>1000	>1000	>1000	>1000	>1000	>1000	>1000	>1000	1000	1000	1000	1000	>1000	>1000
*Klebsiella pneumoniae* ATCC 700603	>1000	>1000	>1000	>1000	>1000	>1000	>1000	>1000	>1000	>1000	>1000	>1000	>1000	>1000	>1000	>1000
*Enterobacter aerogenes* ATCC 13048	>1000	>1000	>1000	>1000	>1000	>1000	>1000	>1000	>1000	>1000	>1000	>1000	>1000	>1000	>1000	>1000

**Table 3:** MIC (μg/mL) and MBC (μg/mL) values of PYED-1 on a panel of Gram-negative and Gram-positive multidrug pathogens.

Bacteria	MIC	MBC	Bacteria	MIC	MBC
*S. aureus* ATCC 29213	16	16	*A. baumannii* ATCC 17978	16	16
*S. aureus* 00717	16	16	*A. baumannii* AYE	16	16
*S. aureus* 00142	16	16	*A. baumannii* 60794	32	32
*S. aureus* 90356	16	32	*A. baumannii* 30031	32	32
*S. aureus* 90159	32	32	*A. baumannii* 30032	16	16
*S. aureus* 90319	16	16	*A. baumannii* 90407	32	64
*S. aureus* 00709	32	32	*A. baumannii* 70120	32	64
*S. aureus* 90623	16	16	*A. baumannii* 62258	16	16
*S. aureus* 90246	32	32	*A. baumannii* 3909	32	32
*S. aureus* 61035	16	16	*A. baumannii* 3990	32	16
*S. aureus* 61486	8	16	*A. baumannii* 4190	16	32
*S. aureus* 64428	16	16	*A. baumannii* NM3	16	32
*E. faecalis* ATCC 29212	4	16	*E. coli* ATCC 25922	128	128
*E. faecalis* 90709	8	16	*E. coli* 90117	128	128
*E. faecalis* 30818	8	16	*E. coli* 01283	128	512
*E. faecium* 90725	8	16	*E. coli* 01029	256	1000
*E. faecium* 90122	8	16	*E. coli* 66570	128	512
*E. faecalis* 90842	8	16	*E. coli* 91947	256	1000
*E. faecalis* 90837	8	16	*E. coli* 90050	256	1000
*E. faecium* 62179	8	32	*E. coli* 65964	128	256
*E. faecium* 63131	8	16	*E. coli* 65026	128	512
*P. aeruginosa* ATCC 27859	128	1000			
*P. aeruginosa* 01018	128	1000			
*P. aeruginosa* 01493	128	1000			
*P. aeruginosa* 00865	64	1000			
*P. aeruginosa* 91631	256	>1000			
*P. aeruginosa* RP73	64	1000			
*P. aeruginosa* 66148	128	1000			
*P. aeruginosa* 66149	128	1000			
*P. aeruginosa* 00445	256	>1000			
*P. aeruginosa* 00173	256	>1000			

**Table 4:** Additive effects of the compound PYED-1 with antibiotics against *S. aureus*.

Bacterial Strain	Combination	MIC^a^ (μg/mL)	MIC^c^ (μg/mL)	FIC
*S. aureus* 00717	PYED-1/gentamicin	16/256	8/16	0.5625
	PYED-1/oxacillin	16/128	8/2	0.5156

MIC^a^, MIC of one sample alone; MIC^c^, MIC of samples in combination; FIC, fractional inhibitory concentration.

**Table 5:** RT-PCR analysis of virulence factors gene expression in *S. aureus* ATCC 29,213 in the presence of PYED-1.

Gene	Description	Fold Change^a^ ± SD	*p* Value
*capC*	Capsule biosynthesis protein C	–2.95 ± 0.13	0.0153
*fnbB*	Fibronectin-binding protein B	–2.11 ± 0.04	0.0139
*clfB*	Clumping factor B	+1.44 ± 0.25	0.1988
*spa*	Surface protein A	–1.51 ± 0.02	0.0213
*lytM*	Peptidoglycan hydrolase	+1.39 ± 0.32	0.2177
*aur*	Aureolysin, zinc metalloproteinase	–1.38 ± 0.22	0.0765
*isaA*	Immunodominant staphylococcal antigen	+4.18 ± 1.24	0.0022
*hla*	Alpha-haemolysin	–10.17 ± 0.02	0.0002
*hlb*	Beta-haemolysin	–21.17 ± 0.01	0.0132
*lukD*	Pore-forming leukocidin	–10.96 ± 0.01	<0.0001
*lukE*	Pore-forming leukocidin	–13.52 ± 0.005	<0.0001
*sea*	Staphylococcal enterotoxin A	–2.46 ± 0.02	0.0115
*agrA*	Accessory gene regulator protein A	–2.29 ± 0.1	0.0146
*sigB*	RNA polymerase sigma factor B	–2.11 ± 0.09	0.0006
*saeR*	Response regulator SaeR	–8.66 ± 0.03	<0.0001

^a^—indicates reduction and + indicates increase.
